# A Porcine Model for Urinary Tract Infection

**DOI:** 10.3389/fmicb.2019.02564

**Published:** 2019-11-21

**Authors:** Thomas Kastberg Nielsen, Nicky Anúel Petersen, Kristian Stærk, Rasmus Birkholm Grønnemose, Yaseelan Palarasah, Lene Feldskov Nielsen, Hans Jørn Kolmos, Thomas Emil Andersen, Lars Lund

**Affiliations:** ^1^Research Unit of Urology, Department of Clinical Research, Odense University Hospital, University of Southern Denmark, Odense, Denmark; ^2^Research Unit of Clinical Microbiology, Department of Clinical Research, Odense University Hospital, University of Southern Denmark, Odense, Denmark; ^3^Department of Cancer and Inflammation, University of Southern Denmark, Odense, Denmark; ^4^Coloplast A/S, Humlebaek, Denmark

**Keywords:** cystitis, porcine model, urinary tract infection, *Escherichia coli*, UPEC, swine

## Abstract

Urinary tract infection (UTI) is the most common bacterial infectious disease with a high frequency of recurrence and the leading cause of septicemia. *In vivo* experimentation has contributed significantly to the present-day knowledge on UTI pathogenesis. This research has traditionally been based on murine models of UTI. Occasional conflicting results between UTI in mice and humans and increasing skepticism toward small rodent models in general warrant the need of novel large-animal infection models that better resemble the anatomy and physiology of humans, and thus better mimic the course of infection in humans. Here, we report, to our knowledge, the first large-animal model of cystitis. The model is based on pigs, and the protocol supports the establishment of persistent, non-ascending infection in this animal and is established without invasive surgical procedures, pain, and discomfort for the animal. The course of infection is monitored by cystoscopy, microscopy of bladder biopsies, and biochemical analysis of urine and blood samples. At termination, harvested whole bladders from infected pigs are analyzed for microbiological colonization using microscopy, histology, and viable bacterial counts. The model is a useful tool in future studies of UTI pathogenesis and opens up novel possibilities to bridge the current knowledge obtained from small-animal UTI models to UTI pathogenesis in humans.

## Introduction

Urinary tract infections (UTIs) are the most common bacterial infection with an annual incidence of community-acquired UTIs of more than 10% for women in the United States (Foxman, 2014). This high incidence is partly explained by the prevalent frequency of recurrent infections following approximately 25–40% of acute UTIs and bringing about severe morbidity in otherwise healthy women (Foxmann, 2010). In Western countries, UTIs also constitute the major part of hospital-acquired infection accounting for 13–19% of cases (European Center for Disease Control, and Prevention [ECDC], 2013; Magill et al., 2014) with a significant risk of progression to urosepsis as is seen in up to 25% of patients (Bjerklund Johansen et al., 2007; Wagenlehner et al., 2008; Tandoğdu et al., 2016).

Treating and preventing UTI is challenging, and the problem is unlikely to diminish in the future. Improved hygiene procedures and antibiotic stewardship are important steps to control UTIs (Cai et al., 2016). However, a better fundamental understanding of UTI pathogenesis is critical to improve treatment regimens and develop more targeted antimicrobials.

Uropathogenic *Escherichia coli* (UPEC) is by far the most frequent uropathogen in both hospitalized patients and in the general population (Foxmann, 2010; Tandogdu et al., 2014). This bacterium and its role in UTI have been investigated extensively over the past decades. Murine models of UTI have been the main tool to elucidate *E. coli* infection pathogenesis (Hung et al., 2009; Carey et al., 2016). Although these studies have provided important knowledge regarding UTI pathogenesis, critical pathogenic mechanisms described in mice are not always consistent with observations done in humans (Hagan et al., 2010; Bielecki et al., 2014; Subashchandrabose et al., 2014; Carey et al., 2016; Stærk et al., 2016). A growing skepticism toward murine models as predictors of human disease in general, further emphasizes the need for large animal models that more accurately reflect pathogenesis and therapeutic effects in humans (Meurens et al., 2012; Perrin, 2014).

Emerging evidence suggests that the immune system proteins in humans are closer related to pig homologs than to mice homologs (Meurens et al., 2012; Seok et al., 2013; Barber, 2016; Dawson et al., 2017). This supports using pigs as intermediate species between rodents and humans to model infection pathogenesis and immune function relevant to humans. The size and anatomical resemblance of pigs and humans also support the use of urological medical equipment such as cystoscopes and urinary catheters, allowing detailed monitoring of the progression of infection and studies of device-associated UTI. The purpose of the current study was to establish a large animal model of cystitis to facilitate the investigation of UPEC pathogenesis and novel therapeutic strategies. We based the infection protocol on a widely used murine UTI model that has been adopted in our laboratory as well (Hung et al., 2009), since this would render possible direct comparison of infection pathogenesis in the two animals in future studies.

A critical step for UPEC to successfully colonize the bladder is the ability to adhere to the epithelial mucosa lining the luminal surface of the urinary tract (Guglietta, 2017). *In vitro* infection experiments with human urothelial cell cultures shows that UPEC establishes as a sessile population on the surface of the epithelium that depends on and leads to upregulation of adhesive fimbriae such as type-1 fimbriae (Stærk et al., 2016; Pedersen et al., 2018). However, bacterial epithelial association and possible biofilm formation during infection *in vivo* is poorly described and has to our knowledge never been studied in humans or large-animal models. This is despite that biofilm production may be a key determinant for the persistence of UPEC in the urinary tract (Soto et al., 2006). Using the porcine model, we inspected the distribution and quantified urothelial-associated bacteria during the course of infection *in vivo*.

The protocol presented supports establishment of a persistent infection in the porcine bladder for prolonged experiments up to 23 days with a continuant substantial colonization of the bladder mucosa. During the infection, the pigs muster a robust inflammatory response. Although the porcine UTI model, compared to a murine UTI model, suffers from reduced scalability, and a higher cost per animal, the model offers novel possibilities to investigate the pathogenic mechanisms and host response of UPEC bladder infection, which should help advancing our understanding of UPEC pathogenesis in humans.

## Materials and Methods

### Bacterial Strains and Growth Conditions

For inoculation, we used UTI89, a human cystitis-derived UPEC isolate of serotype O18:K1:H7, widely used in several *in vitro* and *in vivo* infection assays (Mulvey et al., 2001; Justice et al., 2006; Khandige et al., 2016; Stærk et al., 2016). A green fluorescent protein-expressing variant of UTI89 (hereafter UTI89pMAN01) was used in selected infections to visualize *E. coli* on bladder tissue samples. This strain harbors the pMAN01 plasmid containing a transcriptional fusion of the *gfp*+ gene to the hsp60 promoter, allowing for constitutive expression of the GFP+ protein (Khandige et al., 2016). Before inoculation, UTI89 was pre-cultured to ensure optimal type-1 fimbrial expression, similarly to the murine UTI model protocol (Hung et al., 2009). In short, a single-colony of UTI89 from an overnight (ON) LB agar plate culture was used to inoculate 25 ml of LB broth in a 50 ml tube (Sarstedt) and incubated statically at 37°C overnight. The next day, 25 μl of ON culture was transferred to 25 ml of fresh LB broth and incubated statically at 37°C ON. Immediately before inoculation of the pigs, bacteria were pelleted by centrifugation at 2500 *g* for 20 min, and inoculation suspensions prepared by resuspending bacteria in 0.9% NaCl saline water, adjusting to an optical density of 1.00 at 600 nm and diluting this suspension further 1:10 to reach approximately 1 × 10^8^ colony forming units (CFU)/ml.

### Animals Selection and Experimental Ethics

Twelve female (Landrace/Yorkshire/Duroc) pigs weighing between 35.7 and 38.6 kg were used. The animal protocol established was designed to minimize animal pain and resulted in only mild to moderate degree of discomfort. The pigs were housed in the university hospital animal facility in standardized 3 m^2^ hay-covered pig enclosures and fed with standard feed and *ad libitum* tap water and were allowed to acclimatize and recover from the stress of transportation for 3–5 days preceding the experiments. Experiments were approved by the Danish Animal Experiments Inspectorate, license number: 2017-15-0201-01271.

### Anesthesia

Adequate and correct anesthetization of the pigs are critical to reducing discomfort and stress during inoculation. In addition, inadequate sedation can result in the failure of relaxing the inner and outer urethral sphincters of the pig, thus complicating the catheterization and increasing the risk of injuring the inner-linings of the urethra and bladder, possibly making the pig more susceptible to random infection. Therefore, the anesthetic concentrations should be determined empirically, and from careful observation of vital parameters and signs of wakefulness, adjusted accordingly during the procedure. Pigs were pre-medicated on medetomidine (0.4 mg/kg), midazolam (0.2 mg/kg), and atropine (0.05 mg/kg) injected intramuscularly. Anesthesia was induced and maintained with propofol/fentanyl. After the day of inoculation, routine urine and blood samples were collected in a deep sedation with a mixture of Zolazepam (10,9 mg/ml), Tiletamin (Zoletil 50 Vet, 50 mg/ml), Xylazin (Xyxol Vet, 20 mg/ml), Ketamin (Ketaminol 100 mg/ml), Butorphanol (Butomidor Vet, 10 mg/ml), and Metadon (Comfortan Vet 10 mg/ml) as an intramuscular (IM) injection of 1 ml/10 kg. The sedation was supplemented after 20–30 min with a dose of 1 ml/20 kg, if necessary.

### Transurethral Inoculation

Following anaesthetization, the pigs were placed in a supine position on the operating bed. Both hind legs were raised and moved cranially, and secured with ropes, to expose the urogenital area and making it easier to insert the catheter. After a thorough washing of the urogenital area to remove all visible dirt and debris with warm water and soap, the area was disinfected with two rounds of medical grade iodine solution (7% iodide) application.

Next, the pigs were catheterized with a sterile *Chariérre* 10 silicone foley catheter from which urine samples were collected to determine pre-inoculation CFU count. Blood samples were collected in standard EDTA tubes (BD Vacutainer) from the jugular vein.

Following pre-inoculation sampling of urine and blood, the urine content of the bladder was emptied through the catheter. When the flow of the urine ceased, the remaining urine was removed with a large catheter tip syringe, to ensure that any residual urine did not dilute the bacterial suspension to various extend during the subsequent infection procedure. The drained urine volume greatly varied (50 –500 ml).

**FIGURE 1:**
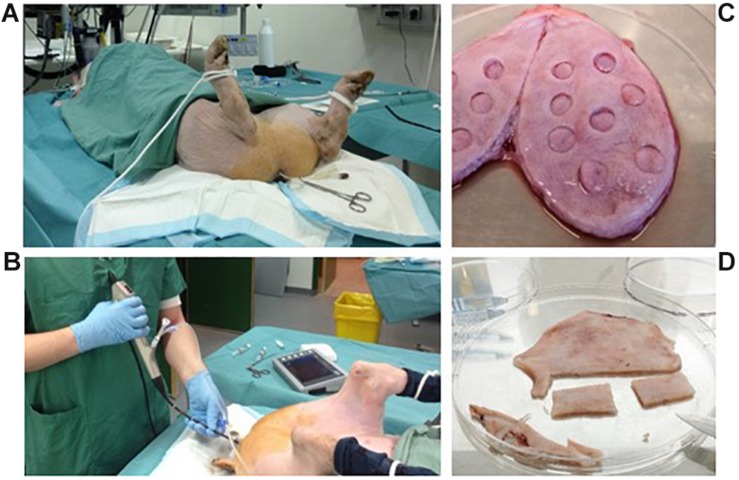
Perioperative conditions and preparation of whole-bladder samples. Inoculation was performed by transferring 100 ml of bacterial suspension through a transurethral catheter and obstructing the catheter with Pean forceps **(A)**. Bladder cystoscopy was performed using a flexible cystoscope **(B)**. After termination of the experiments, samples from harvested whole bladders were prepared for CFU quantification **(C)** or fixed and cut out for microscopy **(D)**.

Two sterile 50 ml syringes, with a catheter tip, containing a total of 100 ml inoculum suspension with approximately 10^8^ CFU/ml were prepared immediately before inoculation (see above). Once the pig was prepared for inoculation, the inoculation suspension was gently infused into the bladder. The process was then repeated with the second syringe. The catheter was then flushed with 10 ml of sterile 0.9 NaCl saline solution, and finally the catheter was clamped using péan forceps (Figure 1A). The inoculum was left in the bladder for 6 h during which the pig was kept on a restricted IV fluid of 0.5 ml/min to control urine production. During this period, the vital signs of the pigs were observed carefully. At the 2- and 6-h mark, urine samples from the catheter, and blood samples from the jugular vein, were collected.

### Collecting Samples and Monitoring the Infection

After inoculation, the pigs regularly underwent cystoscopy and biopsies were collected using a Storz C-VIEW video cystoscope (Figure 1B). Urine was collected through a catheter, and blood sampled every 2 or 3 days. At the same time points, weight-gain, and rectal temperature were measured.

### Sample Analysis

The level of bacteriuria in urine samples were quantified by serial dilution of urine specimens and plating onto LB agar plates (SSI Diagnostica). Blood samples were analyzed for C-reactive protein (CRP) and for interleukin-6 (IL-6) using Enzyme Linked Immuno-Sorbent Assay (ELISA; Pig CRP ELISA Kit (ab205089) and Pig IL-6 ELISA Kit (ab100755), Abcam). Bladder biopsies obtained during infection were analyzed by Confocal Laser Scanning Microscopy (CLSM, see below) and the number of viable bacteria associated with the tissue estimated by plating of homogenates. The latter was conducted by first dipping the tissue specimens sequentially in three different beakers with sterile saline water to wash off loosely adherent bacterial cells. Then, samples were transferred to plastic tubes containing 5 ml 0.9% NaCl saline water, followed by homogenization of the tissue using a rod disperser at 22.000 rpm (IKA Ultra-Turrax T25), serial dilution of the homogenate and plating on LB agar plates (SSI Diagnostica).

### Termination

**FIGURE 2:**
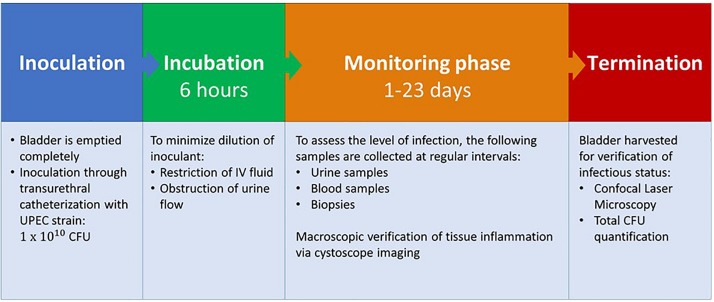
Porcine infection protocol. UPEC, Uropathogenic *Escherichia coli*; CFU, colony forming units.

Pigs were euthanized by sedation with medetomidine 0.04 mg/kg and midazolam 0.2 mg/kg IM followed by 5 mL pentobarbital (200 mg/ml) IV. To yield accurate estimates of bacterial numbers in tissue and urine that were not affected by potential post-mortem acceleration of bacterial growth, bladder, kidneys, and ureters were surgically removed immediately after termination and transferred under sterile conditions to a laminar air flow cabinet for further processing. Bladders were opened by cutting through one side of the bladder wall in between the ureters by using a scalpel (Figure 1C). Smaller specimens (Ø = 1 cm) were punched out of the bladder. Some of these specimens were pinned to a silicone support by using needles and fixed in neutral buffered formalin solution (Sigma) for later histology (see below). Others were used for CFU quantification as described above for biopsies. For fluorescence microscopy and CLSM, whole-bladders were splayed by pinning the bladder in a stretched position onto a silicone support followed by immersion in neutral buffered formalin solution where they were left ON at 4°C. Prior to microscopy, 2 × 4 cm pieces were cut-out and mounted on microscope slides with the surface facing up (Figure 1D). An overview of the complete porcine infection protocol is summarized in Figure 2.

### Confocal Laser Scanning Microscopy

Fixed and mounted tissue samples were either inspected directly with CLSM for visualization of GFP tagged UTI89, or additionally stained to visualize bladder cell cytoskeleton and cell boundaries. For actin cytoskeleton staining, samples were washed in PBS and bladder cell membranes permeabilized for 5 min with 0.5% Triton X-100 (Sigma-Aldrich). The tissue specimens were then stained for 30 min at room temperature with 100 nM Acti-stain 555 phalloidin (Cytoskeleton, Inc.). CLSM was conducted using an Olympus FV1000MPE CLSM equipped with and an Olympus UPlanSApo 20×/0.85 objective. Images were captured using the Olympus FV10−ASW software and brightness and contrast were adjusted with the Photoshop Elements software.

### Histological Examination

Formalin fixated tissue samples from the pig bladder wall were cut in 4 μm sections using a cryostat and stained with van Gieson-Alcian blue to visualize the urothelium and the associated bacteria. Digital images were obtained using an Olympus BX-60 light microscope fitted with an Evolution MP 5.0 color cooled digital camera (Media Cybernetics). Images were analyzed using Image Pro plus 7.0 software (Media Cybernetics, Silver Spring, MD, United States).

### Statistical Analysis

Statistical analysis was performed with GraphPad Prism 8 software version 8.0. Comparisons were performed with one-way ANOVA followed by Brown–Forsythes test for variance and Tukey’s multiple comparisons test. Significance was defined by a *p*-value < 0.05.

## Results

### Significant Bacteriuria Persists in the Porcine Bladder for Prolonged Periods After Inoculation With Uropathogenic *Escherichia coli*

**FIGURE 3:**
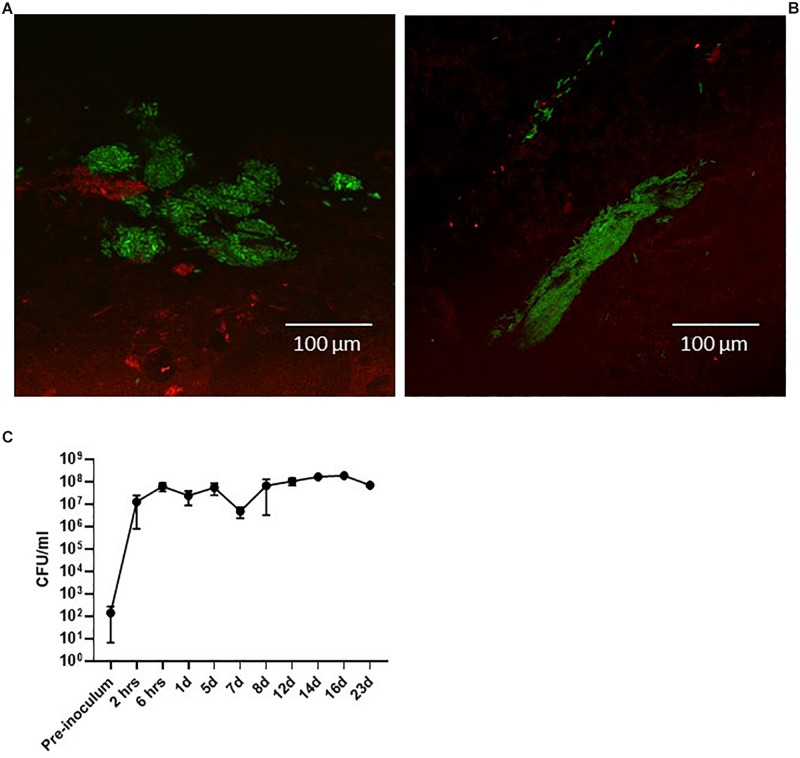
Pigs infected with the green-fluorescent protein-expressing UTI89pMAN01. CLSM of the surface of biopsies performed at 6 h post-infection **(A)** and 24 h post-infection **(B)** revealed bladder-wall associated bacteria. Urine specimens from infected pigs were analyzed to quantify the level of bacteriuria. 6 h after inoculation the pigs have developed significant bacteriuria and remains at this level **(C)**. Graph represents data from six pigs ± standard errors of the mean. Pigs were terminated at 12 days (*n* = 2), 16 days (*n* = 2), and 23 days (*n* = 1). The second pig for the 23-day experiment had to be terminated at 5 days due to illness unrelated to the UTI infection. CFU, colony forming units.

**FIGURE 4:**
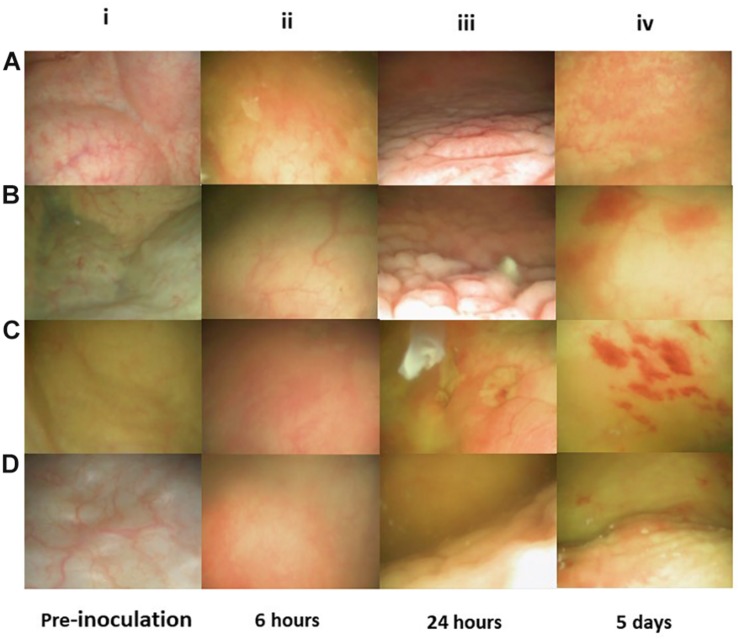
Flex-cystoscopy of pig bladders *in vivo*. At pre-inoculation, the mucosal surface was smooth, uniform in color and with no signs of inflammation **(Ai–Di)**. Six hours post-inoculation (hpi), discrete swelling and reddening of the mucosal surface was observed and the injected liquid became turbid due to cell exfoliation, indicating a state of early inflammation **(Aii–Dii)**. At 24 hpi the mucosal surface was characterized by a high degree edema and inflammation, indicating acute inflammatory reaction **(Aiii–Diii)**. Significantly decreased mucosal edema and reddening was observed at 5 days post-inoculation and instead, scattered spots of haemorrhagic mucosal spots had emerged **(Aiv–Div)**.

An initial question when establishing the model was if the clinical cystitis isolate UTI89 would be able to settle and persist in the porcine bladder after a single inoculation and without any additional intervention. To test this, three groups of two pigs were inoculated with UTI89 and monitored for 12, 16, and 23 days post-inoculation (dpi), respectively. To follow the course of infection, the pigs were sedated biweekly to collect urine and blood samples and to perform cystoscopy and collect biopsies from the bladder. Figure 3 shows that 24 h post-inoculation (hpi), UTI89 is present in the urine in significant numbers averaging at 10^7^–10^8^ CFU/ml (Figure 3C). Analysis of the urine specimens collected at later time points revealed persistent bacteriuria (>10^5^) throughout prolonged experiments up to 23 days, indicating establishment of a chronic infection. To visualize macroscopic signs of local inflammation of the bladder, flexible cystoscopy was performed of the pigs revealing an edematous and inflamed bladder mucosa at 24 hpi (Figures 4Aiii,Biii) indicating an ongoing infection in the bladder, analogous to observations in bladders of women with recurrent UTI (Santoni et al., 2018). Paradoxical to the persistent bacteriuria observed, the bladder mucosal swelling quickly subsided and appeared habitual at 5 dpi except for pinpoint spots of hemorrhage suggesting that the local inflammation eases off during prolonged infection. CLSM of the bladder wall biopsies showed bacteria localized to discrete patches on the bladder epithelial surface (Figures 3A,B), however, most of biopsy samples revealed little to none bacteria. Due to abundant mucus on the urothelium, suitable biopsies containing epithelium tissue were difficult to obtain, and thus, we refrained from this procedure in the following experiments described.

### Inoculation With Uropathogenic *Escherichia coli* Elicits an Acute and Chronic Inflammatory Response in Infected Pigs

**FIGURE 5:**
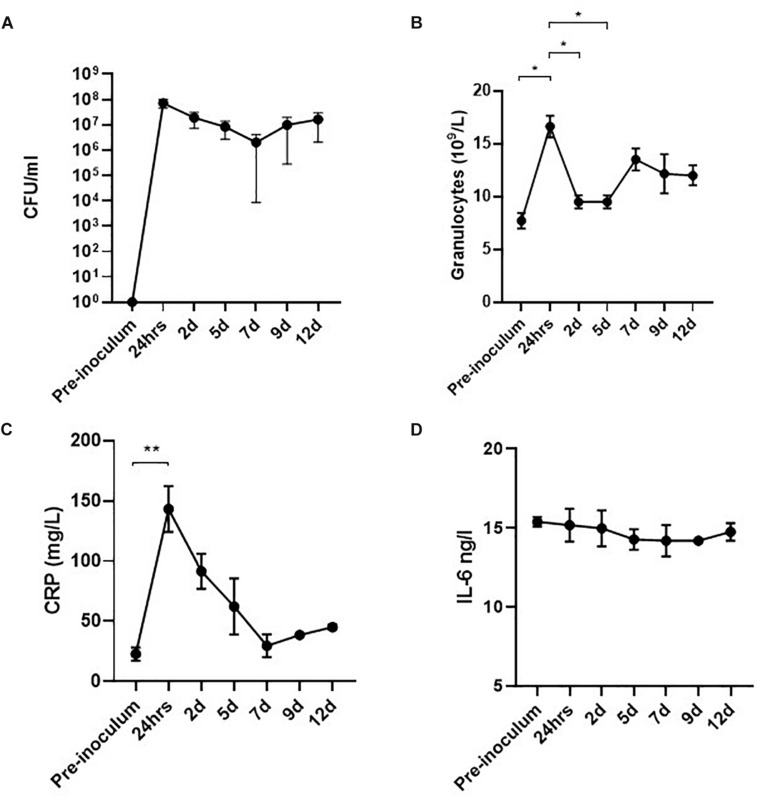
Level of bacteriuria and inflammatory biomarkers during infection of pigs (*n* = 4). The infected pig bladders remain significantly colonized throughout the experiment **(A)**. Within the first 24 h of infection, granulocytes are rapidly rising reaching maximum concentrations of 14.9 × 10^9^/ml. Shortly after, a small drop in granulocyte level is observed though remaining elevated until termination of the experiment **(B)**. CRP increases > 7-fold at 24 hpi (peak-value) and decrease linearly hereafter, reaching baseline values at 7 dpi **(C)**. No observable change in levels of IL-6 was seen during infection **(D)**. Graphs represent means ± standard error of the mean. ^∗^*P* < 0.05, ^∗∗^*P* < 0.001, one-way ANOVA with Tukey’s multiple comparisons test. Data from 7 days onward are based on two pigs only. CFU, colony forming units; IL-6, interleukin-6.

Significant bacteriuria alone is insufficient to define UTI as bacteriuria may represent an asymptomatic colonization of the bladder (Köves et al., 2017). Although the development of fever and cystoscopy images strongly indicated ongoing infection, we further wanted to correlate this with systemic biomarkers of inflammation. In a separate experiment, four pigs were inoculated with UTI89 and monitored for 12 days with urine and blood sample analysis. The results are presented in Figure 5. Within 24 hpi, the pigs developed significant bacteriuria (> 10^7^ CFU/ml) (Figure 5A) as the previous experiment. During the initial 24 h, inoculated pigs responded with rapidly elevated granulocytes levels at an average of 14.9 × 10^9^/L representing a significant 2-fold increase from baseline (*p* < 0.05) (Figure 5B), consistent with an infection of bacterial origin. C-reactive protein (CRP), an acute phase reactant associated with infection in humans, behaved correspondingly with more than 7-fold increase from baseline (*p* < 0.05) (Figure 5C; Wagenlehner et al., 2013). At this time point, the pigs also developed hyperthermia with average temperatures of 39,4°C (± 1,4°C) compared to the average baseline temperature of 36.4°C (±0.8°C) (Supplementary Figure S1). At 2 dpi, granulocytes and CRP-levels declined to sub-maximum values (Figures 5B,C). CRP continued to decline reaching baseline levels at 7 dpi, whereas granulocytes remained moderately elevated throughout the experiment. Altogether, this suggests a progression from acute inflammation into a chronic infectious stage characterized by an attenuated inflammation. This presumption was further supported by the concordance of significantly decreased bladder mucosal swelling at 5 dpi (Figures 4Civ,Div). The inflammatory biomarker, IL-6 was measured in the blood samples as well, but was found to be unaffected during the course of infection which is, however, in accordance with earlier reports from humans (Benson et al., 1994; Roilides et al., 1999).

### UPEC Permanently Colonize the Bladder Epithelial Mucosa

**FIGURE 6:**
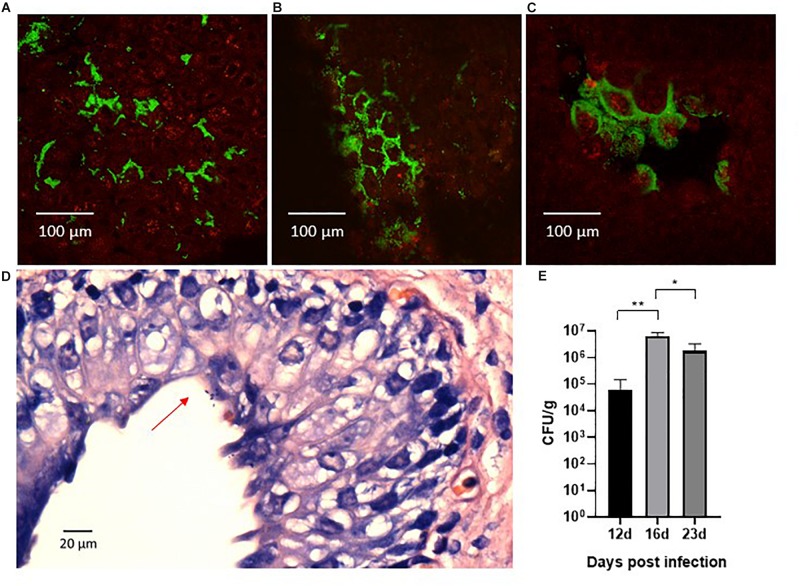
Confocal laser scanning microscopy of the surface of whole bladder sections after 12 h infection with the green fluorescent protein-expressing UTI89pMAN01. Large amounts of bacteria (green) were associated with the bladder tissue (red) although they were found in specific areas of swollen bladder epithelium **(A)**. Large areas in between these infected areas were almost completely devoid of bacteria. The bacteria in infected areas were primarily located in deep pockets of the uroepithelium and inspection at higher magnification revealed that bacteria in general appeared localized to the boarders between the uroepithelial cells **(B)**. At several sites, uroepithelial cells were covered in bacteria that were growing in biofilm-like structures **(C)**. Histological examination revealed surface associated bacteria as well **(D)**, although the above described distribution of bacteria made it difficult with this method to localize colonized sites **(D)**. Whole bladder sections that were washed in PBS, homogenized and plated, showed significant quantities of bacteria associated with the bladder mucosa that persisted for prolonged infection experiments **(E)**. Bars represents means ± standard errors of the mean.^∗^*P* < 0.01, ^∗∗^*P* < 0.0001, one-way ANOVA with Tukey’s multiple comparisons test. CFU, colony forming units.

We sought to investigate the magnitude and morphology of epithelial colonization during infection in our porcine model. Thus, we performed a separate experiment in two pigs that were infected with the green fluorescent protein-expressing UTI89pMAN01 and terminated after 12 h to allow detailed analysis of the progression of colonization at the early state of the infection. Harvested whole bladders from terminated pigs were analyzed for localization and morphology of bacteria using CLSM and histological examination. CLSM showed that large amounts of bacteria were associated with the bladder epithelial mucosa. The bacteria were mainly localized in discrete areas on the bladder luminal surface, rather than distributed randomly on the bladder epithelium (Figure 6A). In between these areas were large areas without any detectable bacteria. Areas with surface-associated bacteria appeared inflamed and were characterized by swollen epithelium with deep pockets that harbored the majority of bacteria in these regions (Figure 6B). At several sites, uroepithelial cells were covered extensively by bacteria that appeared embedded in biofilm (Figure 6C). Histological examination further supported the presence of a surface-associated UPEC (Figure 6D). To quantify epithelial colonization, harvested whole bladder cut-outs from infected pigs were washed in PBS, homogenized, and plated on agar to quantify the number of bacteria associated to the bladder mucosal surface. The results are displayed in Figure 6E showing significant bacterial loads (10^4^–10^7^ CFU/g) at every time-point suggesting persistent epithelial colonization. Homogenates of kidney tissue-sections were sterile (data not shown), confirming that the infection was confined to the bladder without ascending from the urinary tract. Homogenization of ureteral tissue proved infeasible, and thus, no CFU data is available from these samples.

## Discussion

Given that UTI remains a considerable challenge at hospitals and in the community, it is critical that the considerable fundamental biological investigation conducted over the past decades is translated into actual novel treatment regimens. A key step to achieve this is the verification that previous results obtained from *in vitro* and small-animal models also reflect the infection pathogenesis in humans.

Although murine studies have elevated greatly our understanding of general UPEC UTI infection pathogenesis, the general translatability of *in vitro* and murine infection research to human infection is still being questioned in the infection research society. In addition to the major differences in urogenital size and anatomy of mice and humans, and general immune response (Dawson et al., 2017), the highly concentrated nature of mouse urine, compared to the urine of larger mammals such as humans and pigs, is another concern to their frame of reference, since urine concentration is shown to directly influence both UPEC biofilm capacity and UPEC cell morphology, both of which has been linked to virulence in the urinary tract (Parfentjev and Perlzweig, 1932; Justice et al., 2006; Andersen et al., 2012; Klein et al., 2015; Subashchandrabose and Mobley, 2015). In drug development, concerns are also being raised about the use of murine models, as highlighted in a recent report stating that >80% of therapeutic treatments that were safe and effective in animal models, failed to succeed in human trials (Perrin, 2014). These challenges with animal models for understanding pathogenesis and predicting therapeutic outcome in the human host, emphasize the need to improve pre-clinical models to better reproduce the various aspects of disease in humans.

To accommodate this need, we here present a large animal model of cystitis, one of the most frequent infectious diseases in humans. We chose to use pigs as model animal, since they are more similar to humans with respect to urogenital anatomy, urine density, immune system physiology, and furthermore is a natural host of UPEC that recapitulates important aspects of human UTI (Meurens et al., 2012; Barber, 2016). To date, UTI in pigs is limited to a few studies investigating pathology and renal scarring in relation to surgically induced acute pyelonephritis, and to our knowledge, no porcine model exists for establishing a persistent non-ascending UTI (Coulthard et al., 2002; Farhat et al., 2002; Isling et al., 2011; Barber, 2016). Although a non-human primate model of lower UTI with vervet monkeys has been described, a pig model is a feasible alternative due to global availability of animals and lighter ethical concerns (Hyre et al., 2017).

In the porcine model presented here, a single transurethral inoculation with UPEC is sufficient to induce a bladder infection. The pressure generated by transurethral inoculation of 100 ml, could raise concerns about vesicourethral reflux resulting in bacteria reaching the ureters and kidneys, as this has initially proven challenging to avoid in murine models (Schaeffer et al., 1987; Hopkins et al., 1995; Hung et al., 2009). In the porcine model, however, sterile homogenates of kidney tissue samples from infected pigs, indicated that no such bacterial ascension has occurred during inoculation of the pigs. The UPEC strain UTI89 used as infectious agent was originally isolated from a non-ascending cystitis in a human and our results indicate a similar infection symptomatology in the pig (Mulvey et al., 2001). Since the porcine kidney specimens were extracted at the termination of the experiment (>5 days), we can, however, not rule out the possibility that bacteria may have reached the upper urinary tract immediately after inoculation, although insufficient to colonize these sites persistently.

In response to bacterial inoculation, the pigs mustered an inflammatory response of fever, bladder mucosal edema, elevated granulocytes and CRP, all characteristics of UTI in the human host (Wagenlehner et al., 2013; Gupta et al., 2017; Santoni et al., 2018). The onset of ongoing bacteriuria, increased CRP and granulocytes levels also reflect observations from a UTI model with non-human primates (Hyre et al., 2017). After 24 h post-inoculation, the acute infection progressed into a chronic stage characterized by reduced swelling of the bladder mucosa and sub-maximum values of infectious biomarkers. This inflammatory response confirms that the bacteriuria observed is indeed an infection, and not a result of asymptomatic bacteriuria, as is otherwise commonly seen in human patients (Köves et al., 2017). In recent years, the inflammatory cytokine, IL-6, has been investigated as a potential urine biomarker for discriminating between asymptomatic bacteriuria and UTI and has recently been associated with the severity of symptoms in non-febrile lower UTI (Sundén et al., 2017). Increased serum IL-6 levels, on the other hand, has mainly been associated to febrile patients with pyelonephritis (Otto et al., 1999; Gürgöze et al., 2005; Sheu et al., 2006). Also, in studies of neonates and children, urine IL-6 was significantly higher among UTI patients compared to patients without bacteriuria, whereas serum IL-6 did not differ (Benson et al., 1994; Roilides et al., 1999). In the porcine model, no significant change in serum IL-6 was detected during the course of infection, further suggesting that serum IL-6 may be a less promising biomarker compared to urine IL-6 for diagnosing lower UTI. In this study, urine IL-6 was not assessed due to unavailable ELISA reagents. Compared to rodent models, the restricted selection of commercially available porcine reagents is a general limitation of the model to study host-pathogen interactions.

Within the first 24 h post-inoculation, and in agreement with standard diagnostic criteria in humans, the pigs develop significant bacteriuria (> 10^5^ CFU/ml) (Scott et al., 2015). Also, the significant level of bacteriuria persists for prolonged experiments up to 23 days. To survive in the bladder for such a prolonged time, UPEC must be able to successfully circumvent inflammatory host-responses and hydrodynamic flushing by the flow of urine. The latter is likely attributable to a strong epithelial attachment facilitated by various adhesive fimbriae (Connell et al., 1996; Thomas et al., 2004; Jorgensen and Seed, 2012). Here, we show the existence of a significant epithelial colonization *in vivo* with bacterial titers of 10^4^–10^7^ CFU/g. This in agreement with a previous study, in which we showed that during infection *in vitro*, UPEC establishes as a sessile-population on the surface of human urothelial cell layers exposed to a urine hydrodynamic environment (Andersen et al., 2012). Although prone to elimination by urine flushing, bacterial cells released from these sessile populations *in vivo*, may disseminate the infection to uninfected areas of the bladder mucosa as well as other sites of the urinary tract, and this changeable balance between sessile and planktonic growth forms is likely to ensure persistent survival of UPEC in the bladder. Indeed, significant loads of surface-associated bacteria were present in the porcine bladders for prolonged infection for up to 23 days suggesting that the bladder surface is a main habitat for establishing a persistent population in the bladder. Rather than random distribution of individually attached bacteria, CLSM and histological examination revealed that sessile UPEC establish on the bladder luminal surface as small colonies or in biofilm-like aggregates. Prior to this study, the sessile presence and distribution in the urinary bladder has to our knowledge never been assessed *in vivo* and we were surprised to find, that attached bacteria were absent on large areas of the bladder surface. The relatively sparse areas of sessile bacterial presence is a plausible explanation to the lacking bacteria in most biopsied tissue-samples. In future studies, the value of biopsies should be carefully considered, in light of the practical challenges and animal stress involved with the procedure.

In comparison to humans and pigs, mice are extremely resistant to endotoxin shock and responds to infection with hypothermia rather than hyperthermia (Meurens et al., 2012). This is limiting for using mice as models for studying moderate to severe infections associated with hyperthermia. Indeed, a significant characteristic of the porcine-model presented here is the development of moderate fever (39–40°C) during the acute infectious phase. Although fever is not always present in cases of uncomplicated UTI in humans, the absence of bacteria in the kidney tissue homogenates shows that the cystitis isolate UTI89 used here only induces a local infection confined to the porcine bladder, and thus, the fever observed was not due to bacterial ascension in the urinary tract and translocating of bacteria to the blood. This was further supported by the weight gains of the pigs, which were as expected of their age and indicating that they thrived and were most likely not subject to systemic infection and sepsis. It is more likely that the hyperthermia observed was a sign of a massive acute inflammatory process in response to the relatively large bacterial inoculum concentration of 10^8^ CFU/ml. This inoculum concentration was chosen based on what is traditionally used in murine UTI models, where it is required for successful infection, and hence would allow for direct comparison between these two animals (Hung et al., 2009). However, in contrast to mice, pigs and humans are natural hosts of UPEC and naturally susceptible to UTI and therefore this inoculum is presumably larger than necessary for establishing a UTI in these species. Ongoing studies indicate that significantly lower inoculum concentrations are adequate to cause UTI in the pig (data not shown) and hence, could be used in case no direct comparison are made to the mouse. Further studies are needed to uncover the minimal bacterial load necessary for colonizing the porcine urinary tract for optimal comparison to the course of infection in humans, and due to the above reasons, such studies cannot be conducted in rodent models and require large animal models.

Despite the localized infection presented here, the ability of inducing hyperthermia in the porcine model is a quality that grants the potential of studying disseminated infections and urosepsis, a life-threatening complication to infections originating from the urinary tract and one of the most common cause of septicemia (Wagenlehner et al., 2008; Bonkat et al., 2018). To our knowledge, only one study to date, has documented urosepsis in pigs using porcine-isolated pyelonephritis strains, however, in light of the high prevalence of urosepsis in human patients, studies using human pathogenic strains are warranted (Isling et al., 2011).

An extensively studied pathogenic component in UPEC UTI is the type-1 fimbriae (Hagan et al., 2010; Jorgensen and Seed, 2012; Stærk et al., 2016). This adherence fimbrium is critical for murine UTI (Connell et al., 1996; Martinez et al., 2000) and thus believed to play a role in human UTI as well (Connell et al., 1996). Yet, solid evidence is lacking that demonstrates a major role of this fimbria in the pathogenesis of human cystitis (Johnson, 1991; Bower et al., 2005; Bergsten et al., 2007; Jorgensen and Seed, 2012). The down-regulation of type-1 fimbriae in the urine of UTI patients raises questions regarding its significance for human UTI (Hagan et al., 2010; Subashchandrabose et al., 2014) although *in vitro* modeling of infection indicates that this apparent paradox might be explained by expression of the *fim* genes only in surface-associated populations (Stærk et al., 2016).

The inoculation suspension of UTI89 used here, was pre-grown under circumstances that increases type-1 fimbriae expression which is standard protocol used in the murine UTI model to facilitate successful infection (Hung et al., 2009). To which extend this artificial induction of type-1 fimbriae affects the course of infection in the pigs is, however, unknown, but a subject for future research in our group. Previous experimental studies of acute pyelonephritis in pigs showed an increased severity of histological lesions in kidneys after infection with strains expressing both type-1 fimbriae and P-fimbriae suggesting that these fimbriae contribute to UPEC virulence during ascending UTI in pigs (Isling et al., 2011). However, the importance of type-1 fimbriae for non-ascending UTI in pigs remains unknown. Further large-animal studies with type-1 pili attenuated strains are needed to elucidate the significance of this virulent factor during UTI.

In conclusion, a method is presented here that induces persistent cystitis in a large animal model of cystitis thus permitting the study of UPEC pathogenesis in a urinary tract with a physiology comparable to humans. The model development aimed to provide an experimental platform that can help translate decades of extensive UTI research from *in vitro* and murine experiments into clinical therapeutic strategies. The model described, facilitates the study of both acute and chronic UPEC pathogenic mechanisms and host immune effectors relevant to UTI and offers the stepping stone for studying ascending and invasive infections as well. Additional studies in this setup will furthermore enable investigation of current UTI pathogenesis paradigms derived from the murine model, such as intracellular colonization and phenotypic plasticity as possible major determinants of UPEC virulence and persistence (Mulvey et al., 2001; Justice et al., 2008).

## Data Availability Statement

The raw data supporting the conclusions of this manuscript will be made available by the authors, without undue reservation, to any qualified researcher.

## Ethics Statement

This study was carried out in accordance with the recommendations of the Danish Animal Experiment Inspectorate. The protocol was approved by the Danish Animal Experiment Inspectorate, license number: 2017-15-0201-01271.

## Author Contributions

TN, NP, RG, TA, and LL conceived the study idea and design. TN, NP, YP, RG, TA, LN, and LL performed the experimental work. KS completed the first draft of the manuscript. TN and TA contributed to sections of the manuscript. All authors were involved in the discussion and interpretation of the results, contributed to manuscript revision, and has read and approved the submitted version.

## Conflict of Interest

The study was co-financed by Coloplast A/S which also assisted with the histological examinations and interpretation of these results. The conclusions of the study were in no way influenced by the commercial interests of Coloplast A/S. LN was employed by company Coloplast A/S. The remaining authors declare that the research was conducted in the absence of any commercial or financial relationships that could be construed as a potential conflict of interest.
