# Supplementary figures and images for: A Porcine Model for Urinary Tract Infection
Source: Front Microbiol. 2019 Nov 21;10:2564. doi: 10.3389/fmicb.2019.02564 (PMC6882375; doi:10.3389/fmicb.2019.02564)

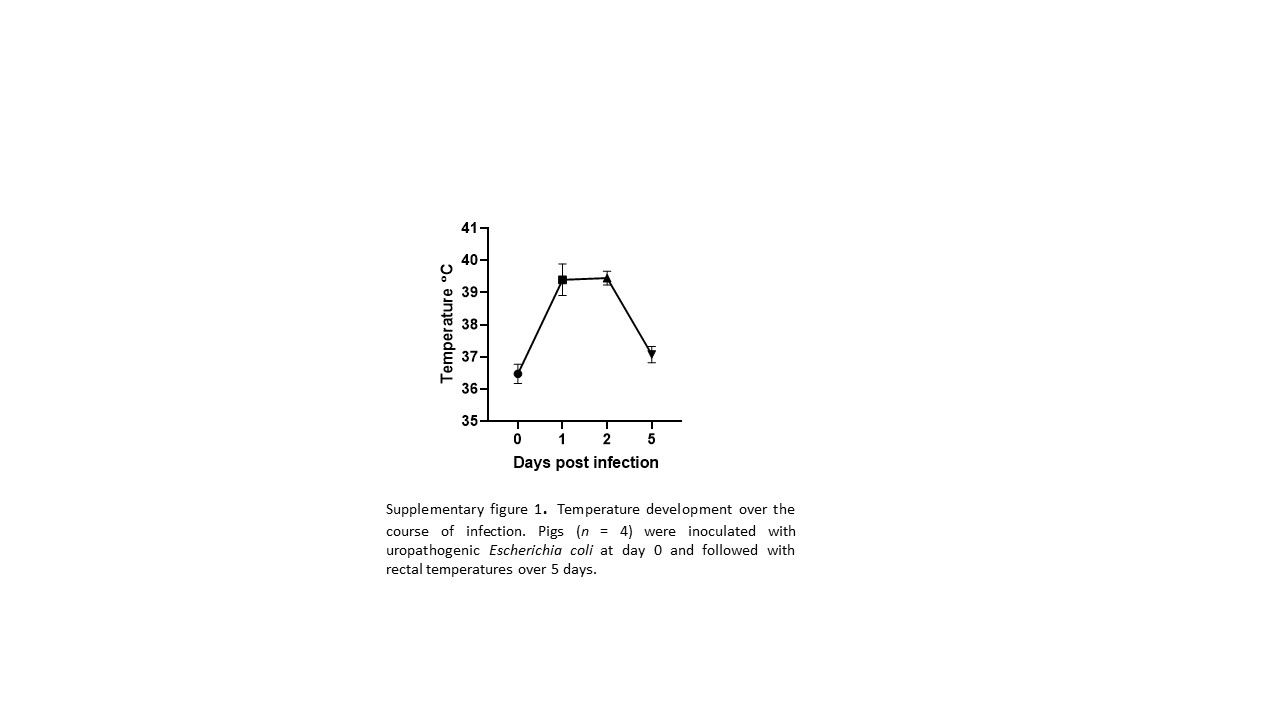

Supplement: Supplementary file 1 [file Image_1.jpg]
